# A Diagnostic Dilemma: Persistent Fever in a Hospitalized Patient With Alcohol Use Disorder and Pneumonia

**DOI:** 10.7759/cureus.77819

**Published:** 2025-01-22

**Authors:** Admire Hlupeni, Ravi Donepudi

**Affiliations:** 1 Immunology, Midlands State University, Gweru, ZWE; 2 Internal Medicine, St. Luke's Hospital, Chesterfield, USA

**Keywords:** alcohol use disorder, alcohol withdrawal seizures, alcohol withdrawal syndrome, clinical institute withdrawal assessment for alcohol‑revised, delirium tremens, persistent fever, pneumonia

## Abstract

Hospitalized patients with alcohol use disorder (AUD) frequently pose diagnostic and therapeutic challenges due to the interplay of substance use, withdrawal, and coexisting medical conditions. This case presents a 49-year-old man with a history of chronic alcohol overuse and previous alcohol withdrawal seizures, admitted for a right middle lobe pneumonia. Initial management included empiric antibiotics, hydration, and nutritional supplementation. He was also placed on symptom-triggered benzodiazepine therapy, guided by the Clinical Institute Withdrawal Assessment for Alcohol-Revised (CIWA-Ar) protocol, to manage alcohol withdrawal. Despite the broad-spectrum antibiotic coverage, the patient continued to experience fever and tachycardia, raising concerns about an atypical infectious process versus the manifestation of severe alcohol withdrawal. Further consideration and evaluation ruled out antibiotic-resistant organisms, pneumonia-related complications, and alternative infections such as urinary tract infection, meningitis, and endocarditis. Blood cultures taken on admission remained persistently negative, and procalcitonin levels were significantly down-trending, indicating the resolution of a bacterial infectious etiology. By hospital day 3, the patient began exhibiting agitation, shakiness, and confusion, along with persistent spikes in temperature, heart rate, and blood pressure, raising strong suspicion for delirium tremens (DT). This necessitated escalating benzodiazepine dosage on the CIWA-Ar protocol. By hospital day 9, the patient's symptoms had largely resolved, and he was discharged with ongoing care for AUD and follow-up for a lung nodule incidentally identified on chest imaging. This case emphasizes the need for a systematic diagnostic approach to differentiate overlapping symptoms in AUD patients, particularly in the context of acute infections and withdrawal syndromes. Early recognition and aggressive management of DT are critical to prevent complications and improve outcomes in this high-risk population.

## Introduction

Hospitalized patients with alcohol use disorder (AUD) often present unique diagnostic and management challenges due to the complex interplay between their substance use and coexisting medical conditions [1]. This complexity is particularly pronounced when acute infections, such as pneumonia, coincide with alcohol withdrawal syndrome (AWS). Both conditions can present with overlapping symptoms, including fever, tachycardia, and altered mental status, complicating diagnosis and delaying appropriate management [2,3].

In this report, we present the case of a patient admitted with pneumonia and a history of AUD who developed persistent fevers during hospitalization. The clinical presentation raised concerns about whether the fevers were attributable to pneumonia or AWS. This case underscores the importance of a systematic approach to differentiate between overlapping conditions and highlights the role of interdisciplinary care in managing such patients.

## Case presentation

History of presenting complaint

The patient was a 49-year-old man with a history of chronic alcohol overuse, a seizure secondary to alcohol withdrawal two years ago, and a left lower extremity fracture sustained during that episode. He presented to the emergency department at noon with a three-day history of intermittent fevers, peaking at 40°C. Despite initial management with acetaminophen, his fever persisted on the day of presentation, prompting medical evaluation.

He reported a severe left-sided nosebleed one week prior, treated at a local emergency room without nasal packing. His alcohol consumption averaged six standard drinks daily, including one drink on the morning of presentation, with no significant changes in intake over the past two years.

The patient denied chest pain, shortness of breath, sore throat, cough, abdominal pain, nausea, vomiting, diarrhea, dysuria, flank pain, recent seizures, hallucinations, palpitations, or excessive sweating. He also denied recent exposure to individuals with viral illnesses, hematemesis, or a history of cirrhosis. Apart from his current illness, he described himself as being in generally good health.

Emergency department findings

In the emergency department, the patient's vital signs revealed a temperature of 37.2°C, a heart rate of 102 beats per minute, a respiratory rate of 17 breaths per minute, a blood pressure of 126/86 mmHg, and an oxygen saturation of 99% on room air. His body mass index was 31.28 kg/m². He was fully alert and oriented to person, place, time, and situation. He was also cooperative, displaying an appropriate mood and affect. The rest of the physical examination was unremarkable.

Significant laboratory findings included an elevated D-dimer of 0.91 µg/mL (normal <0.49 µg/mL), thrombocytopenia (platelet count of 81 K/µL), hyponatremia (sodium level of 131 mmol/L), a low carbon dioxide (18 mmol/L), a mildly elevated glucose level (112 mg/dL), an elevated aspartate aminotransferase (107 U/L), and a normal alanine aminotransferase (41 U/L). The anion gap was slightly elevated (17), and the estimated glomerular filtration rate (eGFR, CKD-EPI) was 113 mL/min/1.73 m². Lactic acid was mildly elevated at 2.6 mmol/L (normal ≤2.1 mmol/L). The patient's blood alcohol level was 163 mg/dL (normal <10 mg/dL). A respiratory viral four-plex screen, including influenza A and B, respiratory syncytial virus, and SARS-CoV-2, was negative. The remainder of the laboratory results was grossly unremarkable (Table 1).

**Table 1:** Laboratory results from the testing performed in the emergency department showing predominantly normal findings, except for moderate thrombocytopenia, mild hyponatremia, acidosis, and significantly elevated aspartate aminotransferase with normal alanine aminotransferase

Test	Reference ranges and/or units	Test result
White blood cell	4.3-10.0 K/ul	7.2
Hemoglobin	11.8-14.8 g/dl	12.6
Mean corpuscular volume	82-99 fl	96.5
Platelet count	140-350 K/ul	81
Sodium	137-145 mmol/l	131
Potassium	3.4-5.1 mmol/l	3.5
Chloride	98-107 mmol/l	96
Carbon dioxide	22-30 mmol/l	18
Anion gap	7-16	17
Blood urea nitrogen	7-17 mg/dl	9
Creatinine	0.5-1 mg/dl	0.69
Glucose	74-106 mg/dl	112
Calcium	8.4-10.2 mg/dl	9.8
Total protein	6.5-8.6 g/dl	9.6
Albumin	3.5-5.0 g/dl	4.9
Alkaline phosphatase	38-126 U/L	110
Total bilirubin	0.2-1.3 mg/dl	1.4
Aspartate aminotransferase	14-54 U/L	107
Alanine aminotransferase	<50 U/L	41
Lactic acid	0.7-2.1 mmol/L	2.6

**Figure 1:**
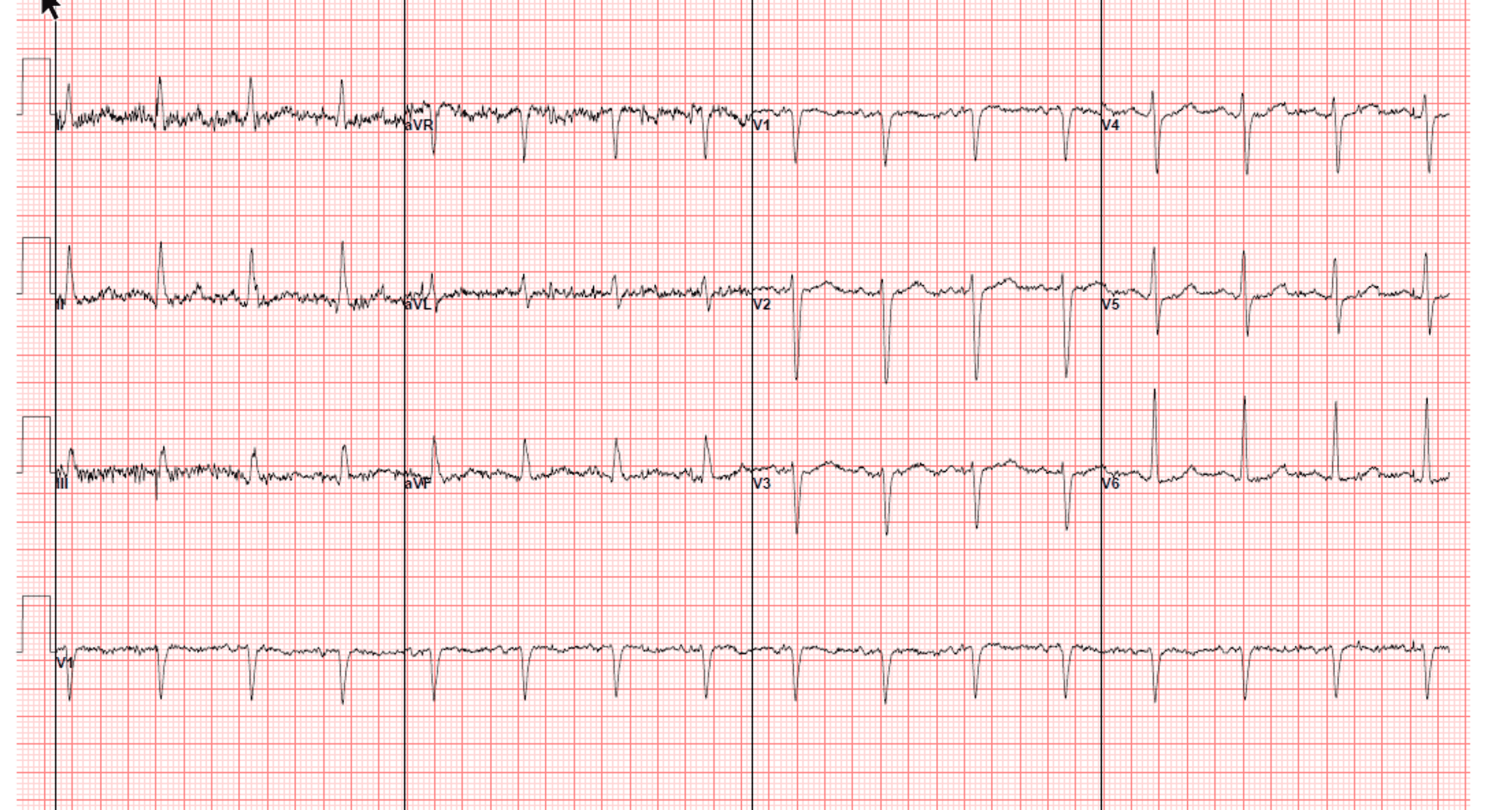
Electrocardiogram taken on admission showing a normal sinus rhythm with a heart rate of 92 beats per minute

An electrocardiogram showed sinus rhythm (Figure 1).

**Figure 2:**
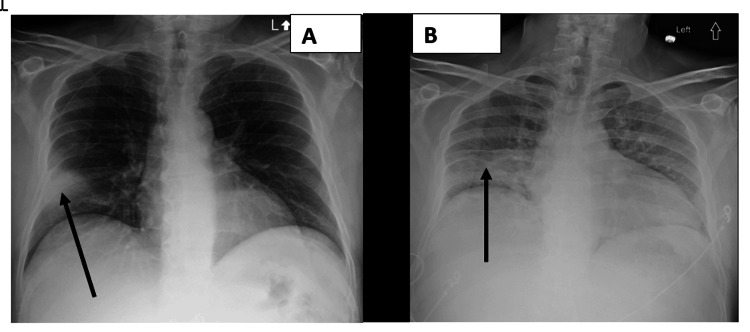
Chest X-rays taken on admission (A) and on day 6 of hospitalization (B). Black arrows indicate areas of consolidation in the right middle-to-lower zones. Both images show no evidence of lung abscess, empyema, or pleural effusion

**Figure 3:**
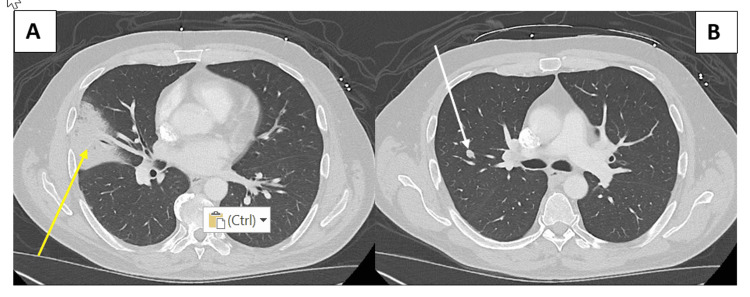
Axial computed tomography images of the chest demonstrating a consolidation in the right mid-lung zone (A, yellow arrow) and a small nodule in the upper right lung (B, white arrow)

Chest imaging demonstrated a right middle lobe consolidation consistent with pneumonia, along with a 0.9 cm nodule in the right upper lobe (Figure 2 and Figure 3).

The patient was administered acetaminophen for fever; azithromycin and ceftriaxone stat doses for pneumonia; lorazepam for alcohol withdrawal prophylaxis; ondansetron for nausea; and thiamine along with folic acid to address potential nutritional deficiencies associated with chronic alcohol use. He also received a liter of normal saline intravenously for hydration. Following this, he was admitted to the medical floor for further investigation and management of his fever in the context of AUD. 

Hospital course

Upon admission, empiric antibiotic coverage was broadened from ceftriaxone to piperacillin-tazobactam. Azithromycin was continued. He was placed on a symptom-triggered benzodiazepine administration using the Clinical Institute Withdrawal Assessment for Alcohol- Revised (CIWA-Ar) protocol to manage alcohol withdrawal. Nephrology was consulted for hyponatremia and acidosis and psychiatry for substance use disorder.

**Figure 4:**
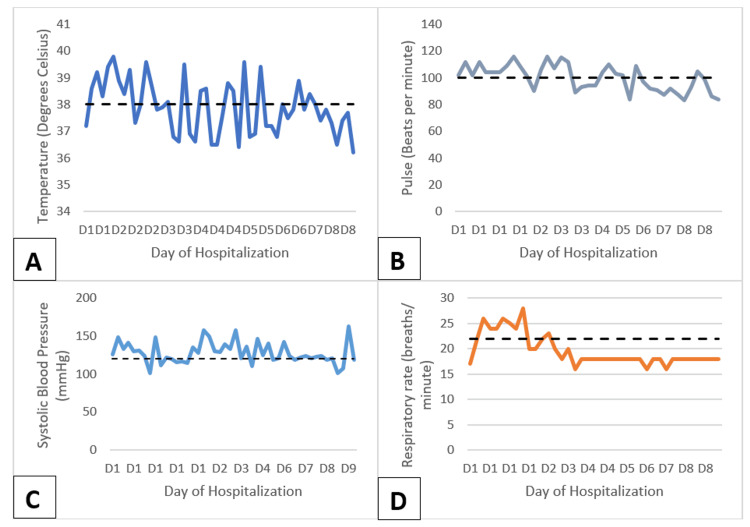
Graphs showing the trends during the patient's hospital stay: spikes in temperature (A), heart rate (B), and systolic blood pressure (C) and the resolution of tachypnea (D)

By the second day, the patient began exhibiting visual hallucinations and made repeated attempts to leave his bed. Spikes of fever and tachycardia persisted. Due to the ongoing fever, pulmonology was consulted to evaluate both the persistent symptoms and the lung nodule incidentally identified on the computed tomography scan. The symptom-triggered CIWA-Ar protocol was continued. The patient was evaluated by the pulmonologist on the morning of day 3. Vancomycin was added to the antibiotic regimen, and methicillin-resistant *Staphylococcus aureus* (MRSA) screening along with a respiratory 22-plex panel was conducted. Despite broadening the antibiotic regimen, the patient continued to experience fever spikes and tachycardia (Figure 4). Later that day, he developed agitation, tremors, and confusion, suggestive of delirium tremens (DT). Management included escalating doses of the benzodiazepine on the CIWA-Ar protocol.

Blood cultures remained negative, and both the MRSA screen and 22-plex returned negative results. As a result, vancomycin was discontinued on day 5. Procalcitonin levels, initially elevated, demonstrated a significant decline with continued treatment, decreasing from 3.56 ng/mL on day 2 to 2.50 ng/mL on day 3 and further to 0.83 ng/mL by day 7. A repeat chest X-ray, despite being of suboptimal quality due to poor cooperation by the patient, showed no significant changes from the initial imaging and did not reveal any evidence of a lung abscess, empyema, or pleural effusion (Figure 2B). By day 9, fever and signs of sepsis resolved, coinciding with clinical improvement in the patient's orientation and near resolution of alcohol withdrawal symptoms.

The hyponatremia (126-132 mmol/L), attributed to syndrome of inappropriate secretion of anti-diuretic hormone likely secondary to pneumonia (on admission: serum osmolality 270 mOsm/kg, urine osmolality 360 mOsm/kg, and random urine sodium concentration 110 mmol/l), persisted despite fluid restriction and electrolyte management. Mild non-anion gap acidosis (venous carbon dioxide 16-20 mmol/L) also persisted, necessitating sodium bicarbonate therapy.

The patient was discharged on day 9 with instructions to continue folic acid, thiamine, and sodium bicarbonate. Follow-up was arranged to address the lung nodule, persistent hyponatremia, mild acidosis, and alcohol rehabilitation. Emphasis was placed on ongoing support for substance use disorder to prevent the recurrence of withdrawal complications.

## Discussion

The persistence of fever (≥38°C), tachycardia (>100 beats per minute), and the development of new-onset altered mentation more than 24 hours after admission in this patient with AUD and confirmed pneumonia, despite empiric broad-spectrum antibiotic therapy, required a comprehensive and multifaceted diagnostic evaluation and step-up of treatment. Several potential explanations were explored.

Immune dysfunction in AUD

Chronic alcohol consumption impairs immune function, predisposing individuals to infections such as bacterial pneumonia through mechanisms like impaired neutrophil function and altered cytokine production [4,5]. While immunosuppression can delay recovery, the patient's response to therapy suggested otherwise. Treatment with piperacillin-tazobactam, azithromycin, and vancomycin, covering a broad spectrum of pathogens, led to stable respiratory parameters and significant down-trending of procalcitonin levels (from 3.56 ng/ml on day 2 to 0.83 ng/ml on day 7), indicating an adequate therapeutic response.

Antibiotic resistance

Antibiotic-resistant organisms, such as *Pseudomonas aeruginosa*, could potentially explain persistent symptoms. However, this patient lacked key risk factors such as recent hospitalization, prior antibiotic use, or chronic respiratory disease [6], making resistance less likely.

Complications of pneumonia

Potential complications, including empyema, lung abscess, or pleural effusion, were ruled out based on stable chest examinations and a repeat chest X-ray on day 6 of admission (Figure 2B), despite suboptimal image quality due to limited patient cooperation.

Alternative sources of infection

Non-respiratory infections such as urinary tract infections, endocarditis, and meningitis were considered. The absence of urinary symptoms, flank pain, headaches, photophobia, or physical signs, such as splinter hemorrhages, Osler's nodes, new heart murmurs, and neck stiffness alongside persistently negative blood cultures, made these diagnoses unlikely. A respiratory 22-plex PCR panel also excluded common viral infections, such as COVID-19 and influenza.

DT

After ruling out infectious and other non-respiratory causes, AWS, specifically DT, emerged as the most probable diagnosis. DT is a life-threatening complication of alcohol withdrawal characterized by confusion, agitation, hallucinations, autonomic hyperactivity (such as fever and tachycardia), and altered mentation. These symptoms often overlap with conditions like pneumonia, complicating timely diagnosis [1-3].

In this case, the patient's history of AUD and recent cessation of alcohol were critical risk factors. Initial symptoms, including anxiety and preserved orientation, which progressed by the third day to vivid hallucinations, confusion, and marked agitation, were consistent with classic DT presentation [7]. Notably, the inability to articulate symptoms due to altered mentation likely delayed the appropriate recognition and treatment of withdrawal symptoms, despite management on a CIWA-Ar protocol.

The patient was stabilized with high-dose benzodiazepines using a symptom-triggered approach; however, scheduled dosing might have been more effective given the severity of symptoms and history of withdrawal seizures [8]. Scheduled dosing ensures consistent suppression of withdrawal-related excitatory neurotransmission and may be particularly beneficial for patients with severe symptoms or impaired communication [8-10]. Adjunctive therapies, such as dexmedetomidine or phenobarbital, were not utilized in this case but could be considered in refractory DT cases [11].

Supportive care played a pivotal role in recovery, including vigilant hydration to prevent dehydration-associated autonomic instability and hyponatremia, vitamin (thiamine and folate) supplementation, and meticulous electrolyte correction to address hypokalemia and hypomagnesemia, which are common in AUD.

## Conclusions

This case report highlights the diagnostic and management challenges in a patient with pneumonia and AUD who subsequently developed AWS. The overlapping symptoms of pneumonia and AWS underscore the need for a comprehensive approach to diagnosis and treatment. When standard infection treatments fail to yield expected improvements, clinicians should maintain a high level of suspicion for AWS in patients with AUD. Early recognition and timely management of AWS, especially DT, are critical for improving patient outcomes and reducing the morbidity and mortality associated with this condition.
